# Spontaneous Expression of Interleukin-2 *In Vivo* in Specific Tissues of Young Mice

**DOI:** 10.1155/1998/12421

**Published:** 1998

**Authors:** Julia A. Yang-Snyder, Ellen V. Rothenberg

**Affiliations:** 1 Howard Hughes Medical Institute and Department of Pharmacology K516 University of Washington Box 357750 Washington Seattle 98195-7750 USA; 2 California Institute of Technology Division of Biology 156-29 Pasadena California 91125 USA

**Keywords:** Dendritic epidermal T cells, gut-associated lymphocytes, TCR-γδ thymocytes, TCR-αβ thymocytes, in situ hybridization, immunohistochemistry, developmental regulation, interleukin-2

## Abstract

*In situ* hybridization and immunohistochemistry were used to determine the spectrum of tissues in which interleukin-2 (IL-2) mRNA and protein are found in healthy, normal young mice. In neonatal animals, IL-2 is expressed specifically by distinct, isolated cells at three major sites: the thymus, skin, and gut. Based on morphology and distribution, the IL-2-expressing cells resemble CD3*ε* ^+^ T cells that are also present in all these locations. Within the thymus of postweanling animals, both TcR*αβ* and TcR*γδ* lineage cells secrete "haloes" of the cytokine that diffuse over many cell diameters. Within the skin, isolated cells expressing IL-2 are seen at birth in the mesenchyme, and large numbers of IL-2-expressing cells are localized around hair follicles in the epidermis in 3-week-old animals. At this age, a substantial subset of CD3*ε* ^+^ cells is similarly localized in the skin. Significantly, by 5 weeks of age and later when the CD3*ε* ^+^ cells are evenly distributed throughout the epidermis, IL-2 RNA and protein expression are no longer detectable. Finally, within the intestine, IL-2 protein is first detected in association with a few discrete, isolated cells at day 16 of gestation and the number of IL-2 reactive cells increases in frequency through El9 and remains abundant in adult life. In postnatal animals, the frequency of IL- 2-positive cells in villi exceeds by greater than fivefold that found in mesenteric lymph node or Peyer's patches. Overall, these temporal and spatial patterns of expression provide insight into the regulation of IL-2 *in vivo* and suggest a role for IL-2 expression distinct from immunological responses to antigen.


*Developmental Immunology*, 1998, Vol. 5, pp. 223–245
Reprints available directly from the publisher
Photocopying permitted by license only

© 1998 OPA (Overseas Publishers Association)
N.V. Published by license under the
Harwood Academic Publishers imprint,
part of the Gordon and Breach Publishing Group
Printed in Malaysia

### Spontaneous Expression of Interleukin-2 *In Vivo* in Specific Tissues of Young Mice

JULIA A.YANG-SNYDER[a] and ELLEN V.ROTHENBERG[b*]

[a]*Howard Hughes Medical Institute and Department of Pharmacology K516, University of Washington, Box 357750, Seattle, Washington 98195-7750;* [b]*California Institute of Technology, Division of Biology 156–29, Pasadena, California 91125*

*(Received 30 May 1997; in final form 20 June 1997)*

*In situ* hybridization and immunohistochemistry were used to determine the spectrum of tissues in which interleukin-2 (IL-2) mRNA and protein are found in healthy, normal young mice. In neonatal animals, IL-2 is expressed specifically by distinct, isolated cells at three major sites: the thymus, skin, and gut. Based on morphology and distribution, the IL-2-expressing cells resemble CD3$\epsilon^+$ T cells that are also present in all these locations. Within the thymus of postweanling animals, both TcR$\alpha\beta$ and TcR$\gamma\delta$ lineage cells secrete "haloes" of the cytokine that diffuse over many cell diameters. Within the skin, isolated cells expressing IL-2 are seen at birth in the mesenchyme, and large numbers of IL-2-expressing cells are localized around hair follicles in the epidermis in 3-week-old animals. At this age, a substantial subset of CD3$\epsilon^+$ cells is similarly localized in the skin. Significantly, by 5 weeks of age and later when the CD3$\epsilon^+$ cells are evenly distributed throughout the epidermis, IL-2 RNA and protein expression are no longer detectable. Finally, within the intestine, IL-2 protein is first detected in association with a few discrete, isolated cells at day 16 of gestation and the number of IL-2 reactive cells increases in frequency through E19 and remains abundant in adult life. In postnatal animals, the frequency of IL-2-positive cells in villi exceeds by greater than fivefold that found in mesenteric lymph node or Peyer's patches. Overall, these temporal and spatial patterns of expression provide insight into the regulation of IL-2 *in vivo* and suggest a role for IL-2 expression distinct from immunological responses to antigen.

*Keywords:* Dendritic epidermal T cells, gut-associated lymphocytes, TCR-$\gamma\delta$ thymocytes, TCR-$\alpha\beta$ thymocytes, *in situ* hybridization, immunohistochemistry, developmental regulation, interleukin-2

### INTRODUCTION

Interleukin-2 (IL-2) has been best studied as a growth factor secreted by activated T cells in the course of the immune response. Its expression is strictly dependent on inducing stimuli, with requirements for separate signals from $Ca^{+2}$-dependent, *ras*-dependent, and possibly additional signaling pathways (Jain et al., 1995; Serfling et al., 1995). These requirements are met in mature T cells when they encounter antigen on antigen-presenting cells, thereby delivering coordinated signals triggered by the crosslinking of TcR/CD3 complexes, CD4 (or CD8 in some cases), and CD28. Accordingly, analysis of the *cis* and *trans*

*Corresponding author.

regulatory elements critical for IL-2 expression has shown several discrete protein-DNA interactions that require activation by TcR/CD3- and/or CD28-dependent signaling mediators (Jain et al., 1995; Serfling et al., 1995). The observation that IL-2 is also expressed primarily or exclusively by T cells has led to a general assumption that the primary function of IL-2 expression *in vivo* is to regulate T-cell clonal expansion as triggered by T-cell recognition of foreign antigen.

This assumption has been challenged in recent years by the phenotype of IL-2 "knockout" mice. Animals homozygous for targeted disruption of the IL-2 gene show initially normal numbers of T cells of various subsets and detectable but relatively subtle defects in their immediate proliferative responses to antigen (Schorle et al., 1991; Kündig et al., 1993; Krämer et al., 1994; Cousens et al., 1995; Kneitz et al., 1995). By contrast, multiple aspects of their long-term hematopoietic regulation and lymphocyte homeostasis are disrupted (Sadlack et al., 1993, 1995; Krämer et al., 1995; Reya, 1996).

These findings raise several questions about the role of IL-2 expression *in vivo*. First, is IL-2 expressed in homeostatic contexts as well as in acute immune responses? Second, does IL-2 expression regulate hematopoietic populations in nonlymphoid tissues? Lastly, is mature cell behavior affected by prior exposure or lack of exposure to IL-2 during development? In order to address these questions, we systematically examined various organs of normal young mice to determine where IL-2 is detectably expressed. Our studies show that IL-2 mRNA and protein are associated with specific tissues *in situ*. These tissues invariably contain T cells, suggesting that IL-2 may be involved in regulating lymphocytic and/or hematopoietic function(s) at these sites.

### RESULTS

### Overall Distribution of IL-2-Expressing Cells in Neonatal Mice

To survey the range of tissues that might express the IL-2 gene in mice, neonatal mouse body sections were analyzed by *in situ* hybridization with $^{35}$S-labeled probes. The results of these analyses showed that most tissues of the body were devoid of hybridization above background, as established with sense-strand control probes. Brain, heart, lung, liver, stomach, bladder, and other parts of the body showed no evidence of IL-2 mRNA at this time (Yang-Snyder, 1994; data not shown). However, cells appearing to express IL-2 were seen reproducibly in three distinct domains: (1) the thymus, (2) the dermal region of the skin, and (3) the intestine. In neonatal animals, the intrathymic IL-2-producing cells appeared as individually labeled cells located predominantly in the inner cortical region, as described previously (Yang-Snyder and Rothenberg, 1993). In the skin and gut, cells expressing IL-2 mRNA appeared as discrete, round cells, as shown in Figures 1(A) to 1(C) (and data not shown). Most IL-2-expressing cells in the skin were located within the mesenchyme, and IL-2-expressing cells in the intestine were found in the lamina propria of the villi. IL-2 protein was also readily detectable in the thymus and gut of neonatal animals (see what follows). Due to technical difficulties, the presence of IL-2 protein in the skin was not determined. Altogether, these data demonstrate that IL-2 mRNA and protein are found in specific lymphoid and non-lymphoid tissues in healthy, normal neonatal mice.

### IL-2-Expressing Cells in Tissues of Weanling Mice

Based on these findings described IL-2 expression in the thymus, skin, and intestine of older animals was examined. *In situ* hybridization of isolated tissues from older weanling mice (3-4 weeks after birth) revealed that IL-2 mRNA$^{+}$ cells continued to be detected in the thymus, skin, and gut (Yang-Snyder and Rothenberg, 1993; Figure 2 and data not shown). Likewise, IL-2 protein was also detected in these tissues at this stage (Yang-Snyder and Rothenberg, 1993; Figures 3 and 4). IL-2 expression in the thymus and gut remained relatively similar to that seen in neonatal animals, whereas IL-2 expression in the skin exhibited a marked spatial pattern of expression that changed according to the developmental age. These

Importantly, the IL-2-reactive "zones" were specific, as this staining was blocked by an excess of recombinant murine IL-2 [Figure 5(D)]. These data suggest that IL-2 secreted by individual cells can diffuse over many cell diameters *in vivo*, and that a prefixation step allows this extracellular IL-2 to be retained and detected as immunoreactive zones.

Many of the immunoreactive zones were found at the cortical-medullary border or apparently within the medulla, where the most mature thymocytes are concentrated. Furthermore, no extensive zones of IL-2 immunoreactivity were detected in thymi taken from SCID or RAG-$2^{-/-}$ mice that lack $TcR^+$ cells, even with prefixation (data not shown). This finding raises the possibility that cells responsible for secreting the large haloes of IL-2 are relatively mature $TcR^+$ thymocytes. To test this interpretation, immunohistochemical staining was performed on sections of thymic tissue from mice homozygous for targeted disruptions in one or more TcR genes, in which development of the $\alpha\beta$-$TcR^+$ and/or $\gamma\delta$-$TcR^+$ lineage is blocked at characteristic checkpoints. These lesions cause significant organizational changes in the thymus, as shown in Figure 6. Disruption of $TcR\alpha$ interferes with positive selection of $\alpha\beta$-$TcR^+$ T cells and suppresses formation of the medulla, as revealed by the absence of regions of decreased cellularity within the thymus [Figure 6(A)]. On the other hand, disruption of $TcR\beta$ blocks an early stage in the development of $\alpha\beta$-$TcR^+$ cortical thymocytes and essentially eliminates the normal structure of the densely populated thymic cortex, yielding a relatively uniform distribution of cells within the thymus and reduced organ size [Figure 6(B)]. Disruption of $TcR\delta$ eliminates the minority $\gamma\delta$-TcR lineage and has less obvious effect on overall thymic architecture and size [Figure 6(C)]. Finally, disruption of both $TcR\beta$ and $TcR\delta$ genes blocks the generation of both $\alpha\beta$- and $\gamma\delta$-$TcR^+$ T cells, yielding a greatly shrunken organ [even more so than that observed in $TcR\beta^{-/-}$ animals; Figure 6(D)].

Although the structural context varied depending on the stage at which thymocyte development and/or maturation was blocked, zones of IL-2 immunoreactivity were observed in thymi taken from animals homozygous for disruption of any single TcR gene [Figures 6(A) to 6(C)]. However, zones of extensive IL-2 protein diffusion were absent in the thymi of mice with disruptions in both the $TcR\beta$ and $TcR\delta$ genes [Figure 6(D)], in good agreement with the data obtained using SCID and RAG-$2^{-/-}$ mice (data not shown). Thus, the production of large amounts of secreted IL-2 protein in the thymus depends on the maturation of at least one lineage of $TcR^+$ thymocytes, but the presence of either $\alpha\beta$- or $\gamma\delta$-$TcR^+$ cells could suffice.

### Variable Persistence and Localization of IL-2-Expressing Cells in Nonthymic Tissues of Postnatal Mice

As mentioned previously, IL-2-expressing cells associated with the skin were found predominantly in the underlying mesenchyme in newborn mice. However, at 3 weeks of age, IL-2 $mRNA^+$ cells in the skin were observed in large clusters, as shown by *in situ* hybridization to ear epidermal sheets [Figure 2(A); data not shown], and appeared to be associated with hair follicles [residual hair shafts were observed in some fields, or round pigmented "pits" in others, as indicated by arrowheads in Figure (2)]. Notably, this expression did not persist at stable levels into later life. By 5 weeks of age, IL-2 RNA was no longer detectable in such clusters [Figure 2(C)], and remained undetectable at least through 14 weeks of age [Figure 2(E)]. Concordant with the mRNA detection, numerous IL-2 immunoreactive clusters were seen in ear epidermal sheets at 3 weeks of age, but decreased markedly by 5 weeks of age, and were not readily detected at all at 14 weeks of age (Figure 3). The observed staining was shown to be specific, as preincubation of the primary antibody with recombinant-derived murine IL-2 abolished staining altogether (data not shown). The absence of immunoreactive clusters in epidermal sheets taken from older animals could not be attributed to a problem with retaining immunoreactive protein in the samples, since prefixation of ear epidermal sheets in paraformaldehyde did not increase the sensitivity of staining in samples taken from mice at any age (data

TABLE I Frequencies of IL-2-Reactive Cells Detected by Immunohistochemical Staining of Normal Small and Large Intestine Sections[a]

| Sample | Number of IL-2+ cells | Number of nuclei counted | Percentage of IL-2+ cells |
|---|---|---|---|
| Small intestine (villi): 3 weeks | 220 | 9544 | 2.3 |
| Large intestine: 3 weeks | 112 | 9451 | 1.2 |
| Small intestine (villi): 3 months | 429 | 9629 | 4.5 |
| Large intestine: 3 months | 121 | 9378 | 1.3 |
| Peyer's patch: 3 months | 47 | 5883 | 0.7 |
| Mesenteric lymph node: 3 months | 22 | 3505 | 0.6 |

[a]Following immunohistochemical staining, sections were counterstained with DAPI. Nuclei were visualized under fluorescence microscopy. Random fields at 200× magnification were analyzed to remove bias toward positively staining areas. No immunoreactive cells were observed in negative control stainings of serial sections.

not shown). Thus, the expression of IL-2 by isolated cells in the dermis of neonates appears to progress to synchronous secretion of IL-2 by clusters of epidermal-associated cells at 3 weeks of age, followed by a shutoff of IL-2 expression in subsequent weeks.

In the intestinal tract, expression of IL-2 persisted in a stable pattern in neonates and older animals (Figure 4). Although isolated positive cells were observed by *in situ* hybridization of intestine samples taken from postweanling mice using $^{35}$S-labeled probes, nonspecific background as determined with the sense-strand control probe also increased significantly after birth (data not shown). Additionally, IL-2 protein was difficult to detect in the gut using conventional fresh-frozen sections (data not shown), but sensitivity of staining was enhanced greatly by prefixation of the tissue in paraformaldehyde (Figure 4). In neonatal animals, the percentage of cells positive for detectable IL-2 protein was always significantly higher (~20-fold) than the cells with detectable IL-2 mRNA (data not shown). This did not reflect a lack of specificity in the immunohistochemical staining, but rather the increased persistence of the IL-2 protein as compared to the mRNA within the cell of origin and/or when associated with IL-2-binding target cells.

In weanling and postweanling animals, cells in both the small and large intestine were found to be immunoreactive to IL-2 antibody at relatively constant frequencies from 3 weeks to 3 months of age (Figure 4). In these young adult animals, most intestinal staining was observed in lamina propria-associated cells as opposed to intraepithelial cells. Once again, the specificity of this staining was shown by antibody blocking using recombinant-derived murine IL-2 (Figure 4, right-hand panels). The frequency of IL-2 immunoreactive cells in the lamina propria was actually higher than the frequency of positive cells found in specialized lymphoid tissues, such as Peyer's patches and mesenteric lymph nodes (see Table 1), indicating that IL-2 expression in the gut is found predominantly in "nonlymphoid" sites in the gut.

### Association of IL-2 Expression with the Presence of Mature T Cells in the Skin and Gut

In the thymus, the vast majority of cells are within the T-cell developmental lineage, and the morphology of IL-2 RNA+ cells as already reported indicates that these cells are part of the T-lineage majority rather than part of the minority of morphologically distinct stromal cells (Yang-Snyder and Rothenberg, 1993). T-lineage cells from the skin and gut have also been shown to express IL-2 when activated by immunological stimuli (Gramzinski et al., 1993; Matsue et al., 1993b; Guehler et al., 1996; Lundqvist et al., 1996) However, the connection between the developmental patterns of IL-2 production observed in these tissues and the presence of T cells remained to be defined. To determine whether IL-2 producers might colocalize with T cells in the skin and gut, ear epidermal sheets and gut sections were stained for the expression of T-cell markers.

In the skin, the distribution of IL-2 producers in follicle-associated clusters did not obviously correlate with any previous report of dendritic epidermal T-cell distribution (Miyauchi and Hashimoto, 1989; Payer et al., 1991; Elbe et al., 1992), albeit the sharp age dependence of the clustered IL-2 expression raised the possibility that T-cell distribution at this critical time point had not been examined. Therefore, immunohistochemical staining analyses were carried out using an antibody specific for the invariant CD3$\epsilon$ component of the TcR. Staining of ear epidermal sheets from 5-week-old mice with anti-CD3$\epsilon$ yielded a familiar dendritic pattern (data not shown), but at 3 weeks of age, clusters of heavily stained cells as well as the expected "dendritic" pattern were observed [Figure 7(E)]. The distribution of CD3$\epsilon$ staining was not due to an epidermal organization artifact, since it was highly dependent on age and the presence of mature T cells (see what follows). Importantly, this pattern resembled markedly the pattern of IL-2 protein expression in ear epidermal sheets at the same time [Figure 7(G)]. It should also be noted that occasional, isolated IL-2 mRNA$^+$ cells with dendritic morphology were observed in ear epidermal sheets at 3-4 weeks after birth (data not shown). Side-by-side *in situ* hybridization and CD3$\epsilon$ staining of single ear epidermal sheets taken from 3-week-old mice revealed that when maximal IL-2 expression was observed in this tissue, IL-2 mRNA$^+$ clusters were associated with greater than 60% of hair follicles, and up to 75% of hair follicles had concomitant CD3$\epsilon^+$ clusters (data not shown). The coincident staining of CD3$\epsilon$ and IL-2 in follicle-associated clusters was not due to general trapping of antibodies in these structures. Parallel epidermal sheets stained for IL-2 protein and for the IL-2 receptor $\alpha$ chain (CD25) showed no CD25 immunoreactivity at all, even under conditions where staining of CD25$^+$ cells in fetal thymus was readily detected (Figure 8). Therefore, significant overlap can exist between the distribution of IL-2-expressing cells and CD3$\epsilon^+$ cells in the skin at 3 weeks of age, suggesting that the dense clusters of IL-2-expressing cells likely contain substantial numbers of CD3$\epsilon^+$ cells.

CD3$\epsilon^+$ cells in the gut were also readily detected in the regions where IL-2-expressing cells were found. The percentage of CD3$\epsilon^+$ cells of all classes was found to be modestly higher than the percentage of cells staining for IL-2 protein in any given domain (7.3% and 3.5% CD3$\epsilon^+$ cells in the small and large intestine, respectively, as compared with 4.5% and 1.3% IL-2 protein-positive cells in these domains at 3 months of age), and at least 20-fold higher than the percentage of cells with IL-2 RNA (data not shown). Both TcR$\alpha\beta$- and TcR$\gamma\delta$-positive subsets were present, with CD8$\alpha$-positive cells present at 50-70% the frequency of CD3$\epsilon^+$ cells overall (Yang-Snyder, 1994). It should be noted that due to the ambiguity in distinguishing IL-2-expressing cells from IL-2-binding cells by this assay, it was not possible to further define the relationship between IL-2 production and CD3$\epsilon$ expression in this region.

### Dependence of *in vivo* IL-2 Expression on the Presence of Mature Lymphocytes

To test whether skin- and gut-associated T cells might be required for the spontaneous expression of IL-2 *in vivo*, wild-type mice were compared with immunodeficient SCID mice that lack mature T or B cells, due to a defect in double-stranded DNA break repair required for the completion of successful TcR and Ig gene rearrangement (Bosma and Carroll, 1991). With respect to all other cell lineages, SCID animals are normal, and they remain fertile and healthy overall. In the skin, the *scid* mutation blocked the appearance of IL-2-expressing cells (Figure 9). Both IL-2 mRNA [Figure 9(C)] and IL-2 protein [Figure 9(E)] were undetectable in ear epidermal sheets from 3-week-old SCID mice. Likewise, clusters of CD3$\epsilon^+$ cells are conspicuously absent in the mutant samples [Figure 9(A)], supporting the identification of these novel assemblages in the skin of normal mice as clusters of mature T cells. These data reveal a strong correlation between IL-2 expression and the presence of mature T cells in the skin.

Similar observations were made for IL-2 expression and the presence of mature T cells in the gut. In contrast to neonatal wild-type gut, IL-2 mRNA$^+$ cells could not be found in the neonatal SCID gut [Figures 10(A) and 10(B)]. With regard to protein production, the SCID gut was also deficient [Figures

### Precocity of "Spontaneous" IL-2 Expression in the Gut

The presence of IL-2 in these tissues raises questions about its functional significance in the context of the stimuli that induce it. Several possibilities can be envisioned. Since IL-2 expression appears tightly associated with the presence of mature T cells and most of the animals studied here were not maintained under sterile conditions, it is possible that the induction of IL-2 expression occurs as part of a conventional immune response. Additionally, since IL-2 is a pleiotropic cytokine with receptors expressed on a variety of cells, including intestinal epithelial cells (Ciacci et al., 1993; Reya, 1996; Reya et al., 1996), IL-2 expression could participate in conditioning the microenvironment, thereby performing a role mandated more, perhaps, by developmental cues than by acute immunological challenge. Finally, since IL-2 is a potent growth factor, IL-2 may play a role in the expansion of T-cell populations, again in a way that might respond to homeostatic population maintenance signals (Rocha et al., 1989) rather than to contact with a foreign antigen.

To help address some of these questions, we examined tissues from mice that had not yet left the womb and could not yet have begun to feed. In such animals, IL-2-expressing cells were not easily detected in the skin (data not shown; see Discussion). However, IL-2 protein was observed in the gut of older fetal animals (day 19-20 of gestation), as shown in Figure 11(A). "Nests" of cells staining positively for IL-2 protein were already concentrated within villi. To establish how early IL-2 might be found in the gut, tissue from younger fetal animals was analyzed in parallel. By day 16 of gestation, rare IL-2 immunoreactive cells were already present in the gut, as shown in Figure 11(B), albeit at a frequency (0.3%) that was about tenfold lower than the frequency at day 19-20 of gestation. Although the origin of these IL-2 producers is not clear, we have previously shown that cells staining strongly for IL-2 protein are present in the fetal liver and omentum as well as the fetal thymus by day 15 of gestation (Reya et al., 1996). Our results suggest that some of the first lymphoid cells immigrating to the fetal gut may be induced to express IL-2 even while in transit. The precocious onset of IL-2 expression in these populations of fetal cells suggests that IL-2 expression is widely induced by developmental cues and not solely by "immunological" responses to encounter with foreign antigens.

### DISCUSSION

The results presented here show that cells are induced to express IL-2 in at least three distinct sites in the young mouse *in vivo*: the thymus, skin, and gut. The regulation of IL-2 expression as a function of developmental state and presumed stimulus varies at each site. Significantly, IL-2 expression in these sites is correlated with the presence of mature lymphocytes, and more particularly with T cells. In contrast, IL-2 RNA and protein are not readily detected in any tissue parenchyma or in the brain, providing further evidence for the general T-cell specificity of IL-2 expression.

An unanticipated feature of the pattern of expression is the abundance of IL-2 production in contexts where the stimulus is unlikely to be a foreign antigen. In addition to the ongoing expression of IL-2 by cells within the thymus, we document two further cases where responses to exogenous antigen or to any clonotypic stimulus seem unlikely to explain the control of IL-2 expression. One case is the skin, where lymphoidlike cells deep in the mesenchyme appear to express IL-2 within hours of birth, and whole clusters of cells later secrete high levels of IL-2 until between 3 and 5 weeks of age, when IL-2 expression stops abruptly. Whatever the trigger for the shutoff, there is no straightforward way to correlate the domains and stages in which IL-2 is expressed by these cells with putative exposure to an environmental antigen. The other case is the intestine, surely a site of exposure to environmental antigens in postnatal life. However, IL-2 expression in the gut first appears at E16 and persists from this point onward; hence, environmental antigen derived from feeding cannot be responsible for stimulating the these primitive fetal cell types, does persist in the adult thymus as well (Hua Wang and E.V.R., unpublished results). Thus, these cases of frankly TcR-independent IL-2 induction set a precedent for the potential polyclonal induction of IL-2 expression by developmentally staged environmental stimuli.

Outside the thymus, IL-2 production appears to be associated with the presence of mature T cells. The mature T cells in the skin and gut of young mice are likely to have distinct origins. Both appear different from the common circulating pools of T cells that populate the spleen and lymph nodes, where in fact we do not detect significant spontaneous IL-2 expression (data not shown). The T-cell population in the skin is heterogeneous, composed of a major subpopulation of $\gamma\delta$-cells of known origin and a minor one of $\alpha\beta$ cells of unknown lineage. The predominant lineage arises from a unique $V\gamma3(5)^+$ class of fetal thymocytes that are generated in a single wave, starting at about E15, and emigrate from the thymus at about E16-17 (Havran and Allison, 1990; Allison, 1993; Haas et al., 1993). As mentioned previously, these cells share a single invariant TcR with a uniform recognition specificity (Havran et al., 1989). This lineage .may have an unusually high sensitivity to IL-2, since it is preferentially expanded in fetal thymocyte populations that are cultured in the presence of high doses of IL-2 (Leclercq et al., 1990, 1992) and its development is especially sensitive to inhibition by antibodies against the IL-2-receptor $\beta$ chain (CD122) (Tanaka et al., 1992). In contrast, T cells entering the gut in fetal life may also be dominated by the use of a single $V\gamma$ segment [$V\gamma5(7)$] (Carding et al., 1990; Haas et al., 1993), but the junctions and recognition specificities of their TcR are quite diverse. At least some of these cells are thought to be of extrathymic origin, but postthymic cells and cells apparently generated *in situ* contribute to the population, and may continue to seed the gut throughout life (Rocha et al., 1994; Lefrancois and Puddington, 1995).

What T cells in the skin and gut have in common is a requirement for extensive postthymic or extrathymic expansion preceding their role in immune surveillance. The total number of these cells that is generated initially can be very small. At the sole time in gestation when $V\gamma3(5)^+$ cells are generated as a small subset of thymocytes, the whole fetal thymus contains only $\sim 10^5$ cells. Therefore, in order to populate the whole epidermis at a density of approximately 6-8 $\times$ $10^2$ cells/min$^2$ in the adult animal, extensive extrathymic expansion of the $V\gamma3(5)^+$ cells is required. IL-2 expression could be a part of a general growth-stimulating program that is utilized by this population to reach the needed cell density in the huge target organ. For the gut-associated lymphocyte population, there is probably continuing cellular input. Even so, the expression and production of IL-2 begins as early as the arrival of the first lymphoid-appearing cells in the gut, and the frequency of IL-2 protein-positive cells increases tenfold over the next 3 days of gestation. The earliest stages when IL-2-positive cells are seen in the gut is concurrent with the appearance of IL-2-producing cells observed previously in the fetal omentum (Reya et al., 1996; data not shown). Thus, IL-2 expression, or a general cytokine production program of which IL-2 expression is a part, may coincide with an initial period of seeding and expansion of T lymphocytes in the fetal gut. Confirmation that some cytokine-driven growth program is essential for the expansion of both cell types comes from the complete elimination of IEL and DETC as well as natural killer cells in mice with a disrupted common cytokine-receptor $\gamma c$ gene, or with a disrupted JAK3 kinase gene that is used for cytokine-receptor signal transduction (Cao et al., 1995; DiSanto et al., 1995; Park et al., 1995).

IL-2 itself is not required as a unique growth factor for either dendritic epidermal T cells or gut-associated lymphocytes, since these populations are found in mice homozygous for a disrupted IL-2 gene. Preliminary observations indicate that IL-2-deficient mice may even possess close to normal densities of $CD3\epsilon^+$ cells in the skin as early as weaning age (J.A. Y-S., unpublished results). The apparently normal generation of skin T cells in the absence of IL-2 can be attributed to their ability to proliferate in response to other cytokines, IL-7 and IL-15, which can be secreted by keratinocytes (Matsue et al., 1993a; Edelbaum et al., 1995). However, the simple presence of these cells does not prove that they are functionally normal. Moreover, the disruption of the IL-2 gene leads to significant dysfunction in the gut and chronic inflammatory bowel disease, which contribute to the early mortality observed for IL-2-deficient mice (Sadlack et al., 1993). The basis for this dysfunction is still under investigation, but it may be a result of the incomplete redundancy of functions of cytokines that share some but not all effects of IL-2 on target populations. There is evidence that IL-2 can participate directly in several kinds of autocrine or paracrine growth-stimulatory loops in the intestinal milieu throughout life (Ciacci et al., 1993; Gramzinski et al., 1993; Fujihashi et al., 1996), and perhaps also play a role in restricting excessive lymphocyte activation (Kneitz et al., 1995). Thus, the regulation of IL-2 expression *in vivo*, whether "immunological" or "nonimmunological," cannot be dismissed as functionally insignificant. The possible induction of IL-2 by nonimmunological signals raises fascinating questions of molecular mechanism for future study.

In conclusion, our studies raise the possibility that IL-2 expression *in vivo* might be controlled predominantly by signals not emanating from the clonotypic TcR. Though IL-2 gene regulation is perhaps the best studied of all lymphocyte responses to antigen stimulation (Jain et al., 1995; Serfling et al., 1995; Rothenberg and Ward, 1996) and the transcription factors that service the minimal enhancer of the IL-2 gene are now securely associated with termini of known signaling pathways emanating from the TcR/CD3 and CD28 cell-surface receptors, the results presented here suggest that additional activation mechanisms exist that may be as fundamental for the overall biological role of IL-2 as are the familiar ones used in the "classical" immune response. From a molecular standpoint, it will be of great interest to determine the cell-surface receptor-ligand interactions responsible for inducing IL-2 expression *in vivo* and whether novel *cis* and *trans* regulatory elements are involved in regulating IL-2 expression. On a cellular level, further characterization of IL-2-producing and IL-2-responsive cells will undoubtedly increase understanding of such "nonimmunological" functions of IL-2 *in vivo*.

### MATERIALS AND METHODS

### Mice

C57BL/6 Tla$^+$ mice, transgenic mice with an $\alpha\beta$-TcR specific for H-2L$^d$ (Sha et al., 1988), and C.B-17-*scid* (SCID) mice were bred and maintained in our own facility. The SCID breeders were carefully selected to maintain a particularly low level of "leakiness" in their progeny, as described previously (Rothenberg et al., 1993). All SCID animals were maintained in an Isotec flexible film isolator (Indianapolis, IN) with autoclaved food, water, and bedding, but without antibiotic treatment. 2C $\alpha\beta$-TcR transgenic animals, a generous gift from William Sha and Dennis Loh, were rederived to be virus-free by embryo transfer, backcrossed to either B10 (Jackson Laboratories, Bar Harbor, ME) or B10.S (Charles River Laboratories, Willmington, MA) genetic backgrounds, and kept on sterile food and bedding. Regular monitoring of our colony verified that animals used were pathogen-free (data not shown). Neonatal animals were taken within 12 hr after birth. Fetal tissues were taken from fetuses removed from pregnant BL/6 Tla$^+$ and SCID mothers using timed matings. The appearance of a vaginal plug was designated as day 0.

### Removal and Processing of Tissues

Activated lymph node and fetal thymus samples were prepared as described in (Yang-Snyder and Rothenberg, 1993). "Adult" thymi were removed from 3-5-week-old weanling animals. Intestinal samples from adult animals were extensively flushed with cold phosphate buffered saline (PBS) before processing. For *in situ* hybridization or detection of cell-surface antigens, separated tissues and freshly decapitated neonatal body samples were washed thoroughly in cold PBS, blotted dry, immediately embedded in Tissue-Tek O.C.T. compound (Miles Laboratories, Kankakee, IL), and flash frozen on dry ice. If samples were not sectioned immediately, they were first stored at $-80°C$. Eight-$\mu m$ cryosections were collected onto poly-L-lysine-coated slides, air dried briefly, and fixed in freshly made 4% paraformaldehyde in PBS for 10 min on ice. Following a wash in cold PBS, slides were air dried completely and stored with desiccant at −80°C prior to hybridization. At least 12 hr before hybridization, slides were thawed, transferred to 70% ethanol, and kept at 4°C.

For detection of extracellular as well as intracellular IL-2 protein, organs were prefixed in 4% paraformaldehyde for 1-2 hr on ice prior to embedding. Following fixation, organs were thoroughly washed with cold PBS, saturated in 30% sucrose in PBS, and embedded in O.C.T. To correlate IL-2 and cell-surface staining of intestinal samples taken from older animals, 3-5-mm pieces of intestine were cut in half; one-half was embedded directly in O.C.T. and the other was prefixed in paraformaldehyde solution before embedding. Following cryosectioning, both unfixed and fixed samples were soaked in acetone for 20-30 min at room temperature, air dried, and stored with desiccant at −80°C.

For preparation of ear epidermal sheets, ears were collected and hair was removed by shaving or using a hair-removal wax strip kit (Sally Hansen/Del Laboratories Inc., Farmingdale, NY). Shorn ears were then floated on 0.5 M ammonium thiocyanate (SIGMA, St. Louis) in 0.1 M phosphate, pH 6.8, and incubated at 37°C for 10-15 min. The outer epidermis was separated from the underlying tissue and washed immediately in cold PBS. Whole ear epidermal sheets from the same age animals were used as pairs in all *in situ* hybridization experiments, with one hybridized to the antisense probe and one to the sense-strand control probe. For the modified "whole-mount" *in situ* hybridization procedure, unmounted epidermal sheets were fixed in 4% paraformaldehyde solution as before, then dehydrated in graded ethanols prior to storage in 70% ethanol at 4°C. For immunohistochemistry and immunofluorescence, epidermal sheets were air dried onto coated slides, fixed in acetone at room temperature for up to 1 hr, and rehydrated immediately in PBS and stained.

### Hybridization Probes

Either 410- or 590-nucleotide (nt) IL-2 antisense or sense RNA probes were used as described (Yang-Snyder and Rothenberg, 1993). Details for the synthesis of $^{35}$S-labeled transcripts are given elsewhere (McGuire and Rothenberg, 1987; McGuire et al., 1988). *In vitro* transcription of digoxigenin-11-UTP (dig)-labeled RNAs was carried out according to the manufacturer's specifications (Boehringer Mannheim Biochemicals, Indianapolis, IN; Ransick et al., 1993). Transcription products were resuspended in 200 $\mu$l of 100 mM DTT and 0.1% SDS and stored at −80°C. $^{35}$S-labeled transcripts were used within a week and dig-labeled probes were used up to 6 months after synthesis.

### *In Situ* Hybridization Using $^{35}$S-Labeled Probes

Whole body and isolated tissue sections were hybridized to $^{35}$S-probes, as described in Yang-Snyder and Rothenberg (1993). Hybridized sections were dehydrated in graded ethanols containing 0.3 M ammonium acetate, dipped twice in NT-2B Nuclear Track Photographic Emulsion (Eastman Kodak, Rochester, NY), and air dried in a lighttight box at room temperature. Slides were then transferred to a plastic VWR slide box containing drierite, and exposed for 14-21 days at 4°C. Slides were developed at 12-13°C, coverglasses mounted, counterstained with hematoxylin and eosin, mounted with coverglasses, and analyzed by bright and dark field microscopy.

### "Whole mount" *In Situ* Hybridization of Ear Epidermal Sheets using Digoxigenin-Labeled Probes

An alternative "whole mount" procedure (Ransick et al., 1993) was adapted for ear epidermal sheets. Unmounted epidermal sheets were rehydrated through graded ethanols, washed in PBS containing 0.1% Tween-20 (PBST), and digested lightly in 2 $\mu$g/ml proteinase K in PBS for 2-4 min at room temperature. Samples were transferred to glycine, refixed in 4% paraformaldehyde in PBS for 20 min at room temperature, rinsed briefly in PBS, and acetylated with 0.3% acetic anhydride in 0.1 M triethanolamine, pH 7. Following another PBST wash, samples were soaked in 0.1 M Tris-glycine, pH 7.4 for 30 min, rewashed in PBST, and prehybridized in hybridization buffer (50% deionized formamide, 10% PEG-8000, 600 mM NaCl, 20 mM Tris-HCl, pH 7.4, 5 mM EDTA, 500 $\mu$g/ml yeast tRNA, 2X Denhardt's, 0.1% Tween-20, 20 mM CHAPS, and 10 mM vanadyl ribonucleoside complexes) for 3-4 hr at 37°C. In the meantime, dig-labeled RNA probes were briefly hydrolyzed in carbonate buffer (60 mM $Na_2CO_3$, 40 mM $NaHCO_3$, pH 10.2) for 2-3 min at 60°C, quickly transferred to an ice bath, and neutralized with an equal volume of 200 mM sodium acetate, 1% acetic acid (v/v), pH 6.0. Probes were then ethanol-precipitated, resuspended in a solution of 20 mg/ml yeast tRNA (500 $\mu$g tRNA per $\mu$g probe), denatured by heating for 7-10 min at 95°C, and then added directly to fresh hybridization buffer to a final concentration of 0.05-0.1 ng/ml hydrolyzed probe. Following prehybridization, samples were rinsed in PBST and transferred immediately to a hybridization buffer-containing probe. Hybridization was carried out for 12-15 hr at 42°C. The ensuing day, samples were washed twice in PBST, once at room temperature and then at 37°C, and digested in 100 $\mu$g/ml RNase A in PBS for 20 min at 37°C. After RNase digestion, samples were washed in PBS at 37°C, 1X SSC at 45°C, 0.1X SSC at room temperature, and finally in PBST at room temperature. All post-hybridization washes were carried out for at least 30 min with agitation.

For detection of dig-labeled probe, samples were blocked in 5% goat serum in PBST for 30-60 min at room temperature and then incubated in a 1:200 dilution of alkaline phosphatase-conjugated sheep anti-dig Ab, Fab fragment (Boehringer Mannheim Biochemicals) in PBST containing 5% goat serum and 5% mouse serum for 1-3 hr at room temperature. Samples were subsequently washed several times in PBST for at least 2 hr at room temperature with shaking, twice briefly in Tris-buffered saline +0.1% Tween-20 (TBST) and twice in alkaline phosphatase buffer (100 mM Tris-HCl, pH 9.5, 50 mM $MgCl_2$, 0.1% Tween-20, 1 mM levamisole). Stained samples were developed in alkaline phosphatase buffer containing 338 $\mu$g/ml nitro blue tetrazolium and 175 $\mu$g/ml 5-bromo-4-chloro-3-indolyl phosphate (NBT and X-phosphate, respectively; both from Boehringer Mannheim Biochemicals) for 3-4 hr at room temperature in the dark. Colorimetric reactions were monitored periodically to avoid overdevelopment and stopped by transferring samples to PBST + 1 mM $Na_2EDTA$. Samples were finally dehydrated in graded ethanols, transferred to 2-propanol, cleared in xylenes, and mounted onto glass slides under coverglasses and observed under bright field microscopy.

### Antibodies

Rat anti-mouse IL-2 mAb (S4B6), phycoerythrin (PE)-conjugated anti-mouse $\alpha\beta$ TcR mAb (H57), and biotinylated hamster anti-mouse CD3$\epsilon$ (500.AA2 and 145-2C11) and $\gamma\delta$ TcR (GL3) mAbs were purchased from Pharmingen (San Diego). Fluorescein (FITC)-conjugated hamster anti-mouse CD3$\epsilon$ (145-2C11) was obtained from Boehringer Mannheim Biochemicals and biotinylated rat anti-mouse CD8$\alpha$ from Becton-Dickinson (Mountain View, CA). Biotinylated rat anti-mouse CD4 (H129.19) was purchased from GIBCO/BRL (Gaithersburg, MD). Biotinylated secondary reagents, rabbit anti-rat IgG and goat anti-hamster IgG, both mouse absorbed, were obtained from Vector Laboratories (Burlingame, CA) and CALTAG Laboratories (San Francisco), respectively. Normal rat IgG was reconstituted at a concentration of 20 mg/ml (Miles Laboratories) and normal mouse serum at 60 mg/ml (Cappel/Organon Teknika Corp., West Chester, PA). Recombinant mouse IL-2 was purchased from Genzyme (Boston).

### Immunohistochemistry and Direct Immunofluorescence Staining

Protocols for immunohistochemical and immunofluorescence staining of nonprefixed sections were followed as given in Yang-Snyder and Rothenberg (1993), except that the avidin-biotin blocking step was omitted, all antibody incubations were carried out overnight at 4°C, and washes were increased to 1-2 hr at 4°C. For staining of prefixed sections to detect IL-2, sections were thawed, rehydrated in PBS, postfixed briefly in 1% formaldehyde in PBS for 1-2 min at room temperature, and washed in PBS and incubated in PBST for 30 min at room temperature. Sections were then blocked in PBS/1% BSA, Fraction V/0.1% $NaN_3$ for 1-2 hr at 4°C, after which they were incubated with primary antibody at a concentration of 2-20 $\mu$g/ml in PBS/BSA/azide for 18-20 hr at 4°C in humidified chambers. For the remaining steps, prefixed sections were treated identically to nonprefixed sections. Following a PBS/0.1% $NaN_3$ (PBS/azide) wash, sections were treated with a secondary staining reagent (5-10 $\mu$g/ml) in a similar manner and rinsed again in PBS/azide at 4°C. Samples were then fixed in 2% paraformaldehyde in PBS for 20-30 min., washed in PBS, and then transferred to 1% $H_2O_2$ in methanol for 40-50 min to quench endogenous peroxidase activity. Histochemical detection was carried out using an avidin-biotin-horseradish peroxidase complex (Vectastain *Elite* ABC, Vector Laboratories) and metal-enhanced diaminobenzidine (600 $\mu$g/ml DAB with 20-30 $\mu$g/ml 1:1 $NiCl_2$ and $CoCl_2$ in 50 mM Tris-HCl, pH 7.5). Following detection, thymus sections were counterstained with methyl green and gut samples with DAPI. Samples were cleared in xylenes and mounted under coverglasses. For blocking experiments, the primary antibody was incubated with an excess of recombinant mouse IL-2 for at least 12 hr at 4°C and potential aggregates were cleared from the solution by centrifugation before use in staining.

### *Acknowledgements*

We are very grateful to Drs. Yoichi Shinkai, Fred Alt, Dawne Page, Stephen Hedrick, William Sha, Dennis Loh, Antonio Bandeiras, and Susumu Tonegawa for certain animals used in the course of these studies and for their generous aid in providing some of the tissues. We also wish to thank Drs. Mitchell Kronenberg, Wendy Havran, and Robert Tigelaar for helpful conversations. This work was substantially made possible by the expert and tireless help of Steven Ing-Wa Shen and Robin Monson Condie, who maintained and carefully screened the immunodeficient and transgenic mouse breeding colonies. We also thank Shirley Pease for her assistance in rederiving the transgenic mice; Drs. Eric Davidson, William Dreyer, and Barbara Wold, for access to critical equipment; Joe Umbro and Richard Gomez, for painstaking reproduction of the photographs; Dr. Susan Ward, for key strategic help with some experiments; and Rochelle Diamond, for valuable advice and support. This work was supported by grants from the USPHS to E.V.R., AI34041, AI19752, and AG13108.

### *References*

Allison J.P. (1993). T cell development. Curr. Opin. Immunol. **5**: 241-246.

Bosma M.J., and Carroll A.M. (1991). The SCID mouse mutant: Definition, characterization, and potential uses. Ann. Rev. Immunol. **9**: 323-350.

Cao Y., Shores E.W., Hu-Li J., Anver M.R., Kelsall B.L., Russell S.M., Drago J., Noguchi M., Grinberg A., Bloom E.T., Paul W.E., Katz S.I., Love P.E., and Leonard W.J. (1995). Defective lymphoid development in mice lacking expression of the common cytokine receptor $\gamma$ chain. Immunity **2**: 223-238.

Carding S.R., Kyes S., Jenkinson E.J., Kingston R., and Bottomly K. (1990). Developmentally regulated fetal thymic and extra-thymic T-cell receptor $\gamma\delta$ gene expression. Genes and Dev. **4**: 1304-1315.

Ciacci C., Mahida Y.R., Dignass A., Koizumi M., and Podolsky D.K. (1993). Functional interleukin-2 receptors on intestinal epithelial cells. J. Clin. Invest. **92**: 527-532.

Cousens L.P., Orange J.S., and Biron C.A. (1995). Endogenous IL-2 contributes to T cell expansion and IFN-$\gamma$ production during lymphocytic choriomeningitis virus infection. J. Immunol. **155**: 5690-5699.

DiSanto J.P., Muller W., Guy-Grand K., Fischer A., and Rajewsky K. (1995). Lymphoid development in mice with a targeted deletion of the interleukin 2 receptor gamma chain. Proc. Natl. Acad. Sci. USA **92**: 377-381.

Edelbaum D., Mohamzadeh M., Bergstresser P.R., Sugamura K., and Takashima A. (1995). Interleukin (IL)-15 promotes the growth of murine epidermal gamma delta T cells by a mechanism involving the $\beta$- and $\gamma_c$-chains of the IL-2 receptor. J. Invest. Dermatol. **105**: 837-843.

Elbe A., Kilgus O., Strohal R., Payer E., Schreiber S., and Stingl G. (1992). Fetal skin: A site of dendritic epidermal T cell development. J. Immunol. **149**: 1694-1701.

Fujihashi K., Kawabata S., Hiroi T., Yamamoto M., McGhee J.R., Nishikawa S., and Kiyono H. (1996). Interleukin 2 (IL-2) and interleukin 7 (IL-7) reciprocally induce IL-7 and IL-2 receptors on $\gamma\delta$ T-cell receptor-positive intraepithelial lymphocytes. Proc. Natl. Acad. Sci. USA **93**: 3613-3618.

Gramzinski R.A., Adams E., Gross J.A., Goodman T.G., Allison J.P., and Lefrancois L. (1993). T-cell receptor-triggered activation of intraepithelial lymphocytes in vitro. Int. Immunol. **5**: 145-153.

Guehler S.R., Bluestone J.A., and Barrett T.A. (1996). Immune deviation of 2C transgenic intraepithelial lymphocytes in antigen-bearing hosts. J. Exp. Med. **184**: 493-503.

Haas W., Pereira P., and Tonegawa S. (1993). Gamma/delta cells. Annu. Rev. Immunol. **11**: 637-685.

Havran W.L., and Allison J.P. (1990). Origin of Thy-1$^+$ dendritic epidermal cells of adult mice from fetal thymic precursors. Nature **344**: 68-70.

Havran W.L., Chien Y.-h., and Allison J.P. (1991). Recognition of self antigens by skin-derived T cells with invariant $\gamma$-$\delta$ antigen receptors. Science **252**: 1430-1432.

Havran W.L., Grell S., Duwe G., Kimura J., Wilson A., Kruisbeek A.M., O'Brien R.L., Born W., Tigelaar R.E., and Allison J.P. (1989). Limited diversity of T-cell receptor $\gamma$-chain expression of murine Thy-1$^+$ dendritic epidermal cells revealed by V$\gamma$3-specific monoclonal antibody. Proc. Natl. Acad. Sci. USA **86**: 4185-4189.

Jain J., Loh C., and Rao A. (1995). Transcriptional regulation of the IL2 gene. Curr. Opin. Immunol. **7**: 333-342.

Kneitz B., Herrmann T., Yonehara S., and Schimpl A. (1995). Normal clonal expansion but impaired Fas-mediated cell death and anergy induction in interleukin-2-deficient mice. Eur. J. Immunol. **25**: 2572-2577.

Krämer S., Mamalaki C., Horak I., Schimpl A., Kioussis D., and Hünig T. (1994). Thymic selection and peptide-induced activation of T cell receptor-transgenic CD8 T cells in interleukin-2-deficient mice. Eur. J. Immunol. **24**: 2317-2322.

Krämer S., Schimpl A., and Hünig T. (1995). Immunopathology of interleukin (IL) 2-deficient mice: Thymus dependence and suppression by thymus-dependent cells with an intact IL-2 gene. J. Exp. Med. **182**: 1769-1776.

Kündig T.M., Schorle H., Bachmann M.F., Hengartner H., Zinkernagel R.M., and Horak I. (1993). Immune responses in interleukin-2-deficient mice. Science **262**: 1059-1061.

Leclercq G., DeSmedt M., and Plum J. (1992). Cytokine production and responsiveness of fetal T-cell receptor V$\gamma$3 thymocytes. Scand. J. Immunol. **36**: 833-841.

Leclercq G., DeSmedt M., Tison B., and Plum J. (1990). Preferential expansion of T-cell receptor V$\gamma$3-positive cells in IL-2 stimulated fetal thymocytes. J. Immunol. **145**: 3992-3997.

Lefrancois L., and Puddington L. (1995). Extrathymic intestinal T-cell development: Virtual reality? Immunol. Today **16**: 16-21.

Lundqvist C., Melgar S., Yeung M.M.-W., Hammarström S., and Hammarström M.L. (1996). Intraepithelial lymphocytes in human gut have lytic potential and a cytokine profile that suggest T helper 1 and cytotoxic functions. J. Immunol. **157**: 1926-1934.

Matsue H., Bergstresser P.R., and Takashima A. (1993a). Keratinocyte-derived IL-7 serves as a growth factor for dendritic epidermal T cells in mice. J. Immunol. **151**: 6012-6019.

Matsue H., Cruz P.D., Jr., Bergstresser P.R., and Takashima A. (1993b). Profiles of cytokine mRNA expressed by dendritic epidermal T cells in mice. J. Invest. Dermatol. **101**: 537-542.

McGuire K.L., and Rothenberg E.V. (1987). Inducibility of interleukin-2 (IL2) RNA expression in individual mature and immature T lymphocytes. EMBO J. **6**: 939-946.

McGuire K.L., Yang J.A., and Rothenberg E.V. (1988). Influence of activating stimulus on functional phenotype: Interleukin-2 mRNA differentially induced by ionophore and receptor ligands in subsets of murine T cells. Proc. Natl. Acad. Sci. USA **85**: 6503-6507.

Miyauchi S., and Hashimoto K. (1989). Thy-1$^+$ dendritic epidermal cells undergo mitosis *in vivo*. J. Invest. Dermatol. **93**: 429.

Park S.Y., Saijo K., Takahashi T., Osawa M., Arase H., Hirayama N., Miyake K., Nakauchi H., Shirasawa T., and Saito T. (1995). Developmental defects of lymphoid cells in Jak3 kinase-deficient mice. Immunity **3**: 771-782.

Payer E., Elbe A., and Stingl G. (1991). Circulating CD3$^+$/T cell receptor V$\gamma$3$^+$ fetal murine thymocytes home to the skin and give rise to proliferating dendritic epidermal T cells. J. Immunol. **146**: 2536-2543.

Ransick A., Ernst S., Britten R.J., and Davidson E.H. (1993). Whole mount *in situ* hybridization shows *Endo16* to be a marker for the vegetal plate territory in sea urchin embryos. Mech. Dev. **42**: 117-124.

Reya T. (1996). The role of interleukin 2 in hematopoiesis. Ph.D. diss., University of Pennsylvania, Philadelphia.

Reya T., Yang-Snyder J.A., Rothenberg E.V., and Carding S.R. (1996). Regulated expression and function of CD122 (interleukin-2/interleukin-15 R-$\beta$) during lymphoid development. Blood **87**: 190-201.

Rocha B., Dautigny N., and Pereira P. (1989). Peripheral T lymphocytes: Expansion potential and homeostatic regulation of pool sizes and CD4/CD8 ratios in vivo. Eur. J. Immunol. **19**: 905-911.

Rocha B., Vassalli P., and Guy-Grand D. (1994). Thymic and extrathymic origins of gut intraepithelial lymphocyte populations in mice. J. Exp. Med. **180**: 681-686.

Rothenberg E.V., Chen D., and Diamond R.A. (1993). Functional and phenotypic analysis of thymocytes in SCID mice: Evidence for functional response transitions before and after the SCID arrest point. J. Immunol. **151**: 3530-3546.

Rothenberg E.V., and Ward S.B. (1996). A dynamic assembly of diverse transcription factors integrates activation and cell-type information for interleukin-2 gene regulation. Proc. Natl. Acad. Sci. USA **93**: 9358-9365.

Sadlack B., Löhler J., Schorle H., Klebb G., Haber H., Sickel E., Noelle R.J., and Horak I. (1995). Generalized autoimmune disease in interleukin-2-deficient mice is triggered by an uncontrolled activation and proliferation of CD4$^+$ T cells. Eur. J. Immunol. **25**: 3053-3059.

Sadlack B., Merz H., Schorle H., Schimpl A., Feller A.C., and Horak I. (1993). Ulcerative colitis-like disease in mice with a disrupted interleukin-2 gene. Cell **75**: 253-261.

Schorle H., Holtschke T., Hünig T., Schimpl A., and Horak I. (1991). Development and function of T cells in mice rendered interleukin-2 deficient by gene targeting. Nature **352**: 621-624.

Serfling E., Avots A., and Neumann M. (1995). The architecture of the interleukin-2 promoter: A reflection of T lymphocyte activation. Biochim. Biophys. Acta **1263**: 181-200.

Sha W.C., Nelson C.A., Newberry R.D., Pullen J.K., Pease L.R., Russell J.H., and Loh D.Y. (1988). Positive and negative selection of an antigen receptor on T cells in transgenic mice. Nature **336**: 73-76.

Tanaka R., Takeuchi Y., Shiohara T., Kitamura F., Nagasaka Y., Hamamura K., Yagita H., and Miyasaka M. (1992). In utero treatment with monoclonal antibody to IL-2 receptor $\beta$-chain completely abrogates development of Thy-1$^+$ dendritic epidermal cells. Int. Immunol. **4**: 487-491.

Yang-Snyder J.A. (1994). Anatomical and developmental patterns of interleukin-2 gene expression in the mouse: Analysis of IL-2 expressing cells and partial characterization of interactions involved in mediating IL-2 expression *in vivo*. Ph.D. diss., California Institute of Technology, Pasadena.

Yang-Snyder J.A., and Rothenberg E.V. (1993). Developmental and anatomical patterns of IL-2 gene expression *in vivo* in the murine thymus. Devel. Immunol. **3**: 85-102.

